# Feasibility of anterior pelvic ring fixation alone for treating lateral compression type 1 pelvic fractures with nondisplaced complete sacral fractures: a retrospective study

**DOI:** 10.7717/peerj.8743

**Published:** 2020-03-16

**Authors:** Kun Shang, Chao Ke, Ya-Hui Fu, Shuang Han, Peng-Fei Wang, Bin-Fei Zhang, Yan Zhuang, Kun Zhang

**Affiliations:** Department of Orthopedic trauma, Honghui Hospital, Xi’an Jiaotong University, Xi’an, China

**Keywords:** Lateral compression type 1, Pelvic fractures, Sacral fracture, Anterior pelvic ring

## Abstract

**Purpose:**

The aim of this study was to evaluate the feasibility of anterior pelvic ring fixation alone for treating lateral compression type 1 (LC-1) fractures with nondisplaced complete sacral fractures.

**Methods:**

Patients with LC-1 type pelvic fractures with nondisplaced complete sacral fractures in the Xi’an Honghui Hospital were screened. Those who underwent surgical treatment for the anterior pelvic ring fractures and conservative treatment for the sacral factures were included in the analysis. The Majeed and Short Form-12 (SF-12) functional scores were used to evaluate these patients.

**Results:**

Of the 123 patients enrolled, 108 (88%) responded to our enquiries regarding the outcome. The mean follow-up period was 18.37 months for the 108 patients who responded. The mean SF-12 functional score was 48.22 ± 9.68. The mean Majeed score was 83.47 ± 9.23, including 52 with excellent, 47 with good, seven with fair, two with poor outcomes. The SF-12 functional and Majeed scores were significantly higher in those aged <45 years or without lower limb injury than in those aged ≥45 years or with lower limb injury (*P* < 0.05).

**Conclusion:**

Acceptable functional outcomes can be obtained for LC-1 pelvic fractures with nondisplaced complete sacral fractures by using anterior pelvic ring fixation alone.

## Introduction

Lateral compression type 1 (LC-1) pelvic fractures represent a broad spectrum of injuries, including minimal ‘buckle’ impaction fractures of the anterior sacrum to comminuted sacral fractures that extend to and through the posterior cortex (Khoury et al., 2008). Surgeons might consider multiple factors when determining whether a fracture of this type would benefit from surgical stabilisation. The most important factor is the surgeon’s assessment of fracture stability. However, evaluating the stability of LC-1 pelvic fractures is difficult, and it is a notable research topic in the field of pelvic stability.

Fracture stability is based on initial displacement shown on static radiographs and displacement observed on post-mobilisation radiographs (Bruce, Reilly & Sims, 2011). However, it is difficult to determine whether LC1 fractures are minimally displaced on radiographs obtained shortly after injury (Burgess et al., 1990; Oransky & Tortora, 2007). In clinical practice, pelvic fracture displacement often tends to be underestimated. It has been reported that the peak compression can be 1.3–2.2 times of the final compression appearing on images obtained in the hospital (Ma et al., 2019). Additionally, 37%–68% of LC-1 fractures are associated with clinically significant instability, and they may require operative treatment (Bruce, Reilly & Sims, 2011; Sagi, Coniglione & Stanford, 2011).

In LC-1 pelvic fractures, most sacral fractures are minimally displaced or nondisplaced (Beckmann et al., 2019). LC-1 fractures with incomplete sacral fractures tend to be stable and can be treated conservatively, but LC-1 injuries with complete sacral fractures may indicate instability of the pelvic ring (Bruce, Reilly & Sims, 2011; Lefaivre, Padalecki & Starr, 2009). As for LC-1 injuries with complete sacral fractures, operative stabilisation may be a good choice (Bruce, Reilly & Sims, 2011; Zwingmann et al., 2019).

However, LC-1 injuries have been treated with anterior fixation only, posterior ring fixation only, or anterior and posterior pelvic ring fixation (Avilucea et al., 2018). Because anterior pelvic ring injuries are usually more severe than posterior pelvic ring injuries and fixation of the anterior pelvic ring can provide enough support against the weight-bearing force, we often choose anterior fixation for LC-1 pelvic factures with complete sacral fractures in our hospital. Thus, the aim of this study was to evaluate the feasibility of anterior pelvic ring fixation alone for treating LC-1 pelvic fractures with nondisplaced complete sacral fractures. We hypothesised that acceptable functional recovery would be achieved with anterior pelvic ring fixation alone.

## Methods

### Ethical statement

This study was approved by the ethical board of Xi’an Jiaotong University (approval number 2018109). The need for informed consent was waived by the Ethics Review Board of Xi’an Jiaotong University.

### Patient selection

From November 2016 to December 2018, 367 patients with pelvic fractures in Xi’an Honghui Hospital were selected. According to the Young-Burgess classification for pelvic fracture stability (Young et al., 1986) and with consideration of the mechanism of injury mechanism in each patient, three experienced chief physicians screened LC-1 pelvic fractures with sacral complete fractures by using pelvic anterior-posterior, inlet, and outlet plain, computed tomography (CT), and three-dimensional imaging.

The inclusion criteria were LC-1 type pelvic fractures with a mature skeleton, patients older than 18 years with a complete sacral fracture (unlike with an incomplete sacral fracture, the fracture completely penetrated the sacrum on plain CT), and the sacral fracture was initially not displaced (displacement was measured on the CT scan). The exclusion criteria were spinal cord injury, severe cognitive impairment, patients older than 80 years, incomplete sacral fracture, and complete sacral fracture with displacement greater than one mm.

### Surgical strategy and operative technique

As for anterior pelvic ring fixation, the method might be affected by the reduction and fixation of the posterior pelvic ring. If anterior reduction was performed well and the fracture could be fixed using a minimally invasive treatment, a cannulated screw was inserted into the pubic ramus. If the reduction was inadequate, the Stoppa approach was used (Ponsen et al., 2006). For the Stoppa approach, a 12–15-cm lengthwise incision was made in the middle of the lower abdomen. Subsequently, one or two plates were placed and fixed unilaterally or bilaterally on the pubic ramus. Moreover, the ilioinguinal approach was used in some patients. A C-arm was used to evaluate reduction of the anterior and posterior pelvic rings. Patients were permitted to walk with crutches at 2–3 weeks postoperatively. If there was no pain, full weight bearing was encouraged.

### Indicators of outcome

We obtained final follow-up function scores from the patients who we were able to contact, which were defined as the responder group, and those who could not return to the hospital for various reasons were defined as the non-responder group. The baseline data including the sex ratio, age, injury severity score (ISS), mechanism of injury, combined injury, and duration of hospitalisation were compared between the two groups to assess the difference between responders and non-responders. The main outcome indicators of this study were the Majeed functional score and Short Form-12 (SF-12) functional score. The Majeed functional score is widely used to evaluate the prognosis of pelvic fractures (Lefaivre et al., 2012), and this scoring system (total 100 points) consists of subscores for pain (30 points), return to work (20 points), sitting (10 points), sexual intercourse (4 points), walking aids (12 points), unaided gait (12 points), and walking distance (12 points) (Majeed, 1989). Values ≥85 are considered excellent, 70–84 good, 55–69 fair, and <55 poor. In addition, the SF-12 (version 2) was used to assess the general function and disability status of patients as a measure of general physiological and mental health. The Majeed and SF-12 functional scores of the responders were obtained and grouped according to known factors that may affect the prognosis of patients to evaluate the difference in functional recovery within the groups.

### Statistical analysis

Measurement data such as age, ISS, and duration of hospitalisation were analysed using the independent two-sample *t*-test. The range of anterior ring injury (unilateral pubic ramus fracture versus bilateral pubic rami fracture), postoperative weight-bearing status (partial weight-bearing, full weight-bearing), age (<45 years, ≥45 years), ISS (<15, ≥15), and presence or absence of injury were analysed in the responder group. The Majeed score and SF-12 functional score for complications (with or without lower limb injury) were also analysed with the independent two-sample *t*-test. Pearson’s chi-square test or Fisher’s exact test was used to analyse categorical data, such as sex, classification of the mechanism of injury, combined injury, and anterior pelvic ring fixation. *P*-values <0.05 were considered statistically significant.

## Results

The data of 123 patients with LC-1 pelvic fracture and complete sacral fracture were obtained from the 367 patients. All 123 patients underwent anterior pelvic ring fixation for the LC-1 type pelvic fractures and conservative treatment for the sacral fractures. Of these, 67 patients were fixed with screws, 56 were treated with open reduction and plate fixation, 53 were treated with the modified Stoppa approach, and three were treated with the ilioinguinal approach.

After contacting patients and their families, 108 responders returned to the hospital for further consultation. No differences in sex, age, ISS, mechanism of injury, and duration of hospitalisation were observed between the two groups (Table 1). Regarding the mechanism of injury, traffic crash was the main cause of injury in 39% and 40% of patients in the responder and non-responder groups, respectively. Regarding associated injuries, soft tissue injury was the most common type of injury in both groups. The responder group was followed up for an average of 18.37 (range 7–22) months, and the non-responder group was followed up for an average of 19.13 ± 10.39 months, but without the final functional outcomes. The times to weight bearing were 2.99 ± 0.93 and 3.27 ± 1.28 weeks postoperatively in the responder and non-responder groups, respectively. We evaluated the clinical healing time based on the radiographic findings, symptoms, and signs, and all fractures healed by the last follow-up. Times to healing were 14.59 ± 1.76 and 14.00 ± 1.36 weeks in the responder and non-responder groups, respectively. However, two patients showed displacement of their sacral fractures by 2 and 3 mm, respectively, upon bone union.

**Table 1:** Comparison of baseline data between responders and non-responders.

	Respondents (*n* = 108)	Non-respondents (*n* = 15)	Statistic (t/*χ*^2^)	*P*
**Age**	50.61 ± 17.11	52.33 ± 12.20	0.376^a^	0.708^a^
**Gender (%)**				
Male	51(47)	7(47)	0.002^b^	0.968^b^
Female	57(53)	8(53)		
**ISS**	20.25 ± 10.26	20.53 ± 9.02	0.108^a^	0.914^a^
**Mechanism of injury (%)**				
Fall from height	41 (38)	6 (40)	0.076^b^	0.963^b^
Motor vehicle crash	42 (39)	6 (40)		
Walking injury	25 (23)	3 (20)		
**Combined injury**				
lower limb fracture	10	3	0.672^b^	0.412^b^
upper limb fracture	6	1	–	1.000^c^
craniocerebral injury	5	1	–	0.550^c^
chest injury	16	3	0.019^b^	0.889^b^
abdominal injury	5	2	–	0.203^c^
soft tissue injury	19	4	0.241^b^	0.623^b^
nerve injury symptoms	6	2	–	0.251^c^
**Anterior ring fixation**				
Closed screw fixation	59	8	0.009^b^	0.925^b^
Open reduction and plate fixation	49	7		
**Length of stay in hospital (days)**	8.56 ± 3.84	9.53 ± 3.38	−0.927^a^	0.356^a^
**Follow-up time (month)**	18.37 ± 7.76	19.13 ± 10.39	0.341^a^	0.733^a^
**Weight-bearing time (weeks)**	2.99 ± 0.93	3.27 ± 1.28	1.023^a^	0.308
**Clinical healing time (weeks)**	14.59 ± 1.76	14.00 ± 1.36	−1.250^a^	0.214

**Notes.**

a *t*-test.

b Chi-squared test.

c Fishers exact test was used to calculate the statistics and *P*-value.

ISS, injury severity score

In 108 respondents, 9 sacral fracture lines passed through Denis zone 1, 27 through zone 1 and zone 2, 51 through zone 2 alone, and 12 through zone 2 and zone 3; 9 fracture lines were located in Denis zone 3 alone.

The average SF-12 functional score of the 108 patients was 48.22, and there was no significant difference in the SF-12 functional score between patients with unilateral pubic ramus and bilateral pubic rami fractures (*P* = 0.676). There was no difference in the SF-12 functional score between patients with partial and full weight bearing (*P* = 0.665). There was no significant difference in the SF-12 functional score between patients with ISS <15 and those with ISS ≥15 (*P* = 0.889). However, the SF-12 functional score was significantly higher in patients younger than 45 years (51.45 ± 9.38) than in those older than 45 years (43.52 ± 8.12, *P* = 0.000). In addition, the SF-12 functional score was significantly higher in patients without lower limb injury (48.95 ± 9.27) than in those with lower limb injury (42.92 ± 11.32, *P* = 0.035).

**Figure 1:**
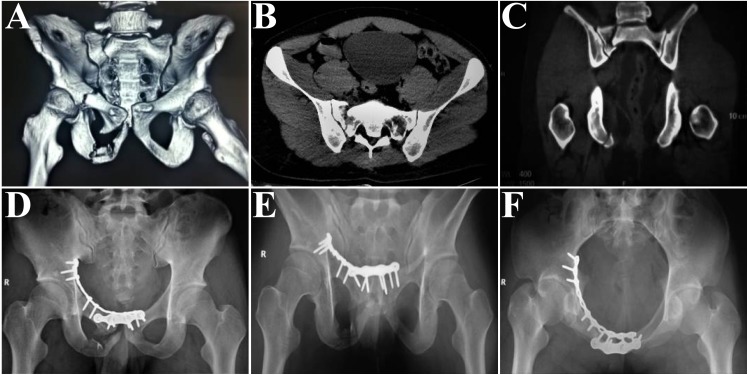
Representative case: a 27-year-old male patient was injured in a traffic crash. The anterior pelvic ring is treated surgically with the modified Stoppa approach and pelvic reconstruction plates for fixation of the anterior pelvic ring. The sacral fractures are treated conservatively. (A) Preoperative three-dimensional reconstruction image, showing unilateral superior and inferior pubic ramus fractures, with right complete, nondisplaced sacral Denis zone 2 fracture. (B) The complete, nondisplaced sacral fracture on an axial computed tomography (CT) scan. (C) The complete, nondisplaced sacral fracture on the coronal CT scan. (D) Postoperative anterior-posterior plain. (E) Postoperative outlet plain. (F) is the postoperative inlet plain.

**Figure 2:**
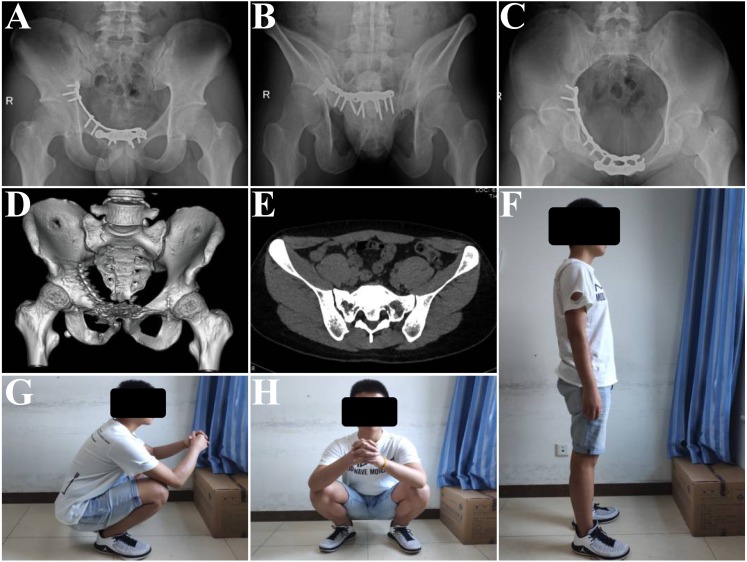
The fracture is united by the follow-up, and the Majeed score is 91 (excellent outcome). (A) The anterior-posterior plain at 5 months postoperatively. (B) The outlet plain at 5 months postoperatively. (C) The inlet plain at 5 months postoperatively. (D) Complete healing of the fracture. (E) Bone union on the axial CT scan; there is no late displacement. (F–H) The patients’ function ability in different positions.

The average Majeed score of the 108 patients was 83.47 ± 9.23. Outcomes were excellent in 52 patients, good in 47, fair in seven, and poor in two. There was no significant difference in the Majeed score between those with unilateral pubic ramus and bilateral pubic rami fractures (*P* = 0.705). There was no significant difference in the Majeed score between patients with partial and full weight bearing (*P* = 0.692). There was no significant difference between patients with ISS <15 and those with ISS ≥15 (*P* = 0.805). However, the Majeed score was significantly higher in patients younger than 45 years (86.52 ± 8.12) than in those older than 45 years (79.05 ± 9.02, *P* = 0.000), with the former having a higher functional recovery. Additionally, the Majeed score was significantly higher in patients without lower limb injury (84.35 ± 8.42) than in those with lower limb injury (77.08 ± 12.41, *P* = 0.007, Table 2). A representative patient is shown in Figs. 1A–1F and Figs. 2A–2H.

**Table 2:** SF-12 and Majeed functional scores of the responders.

	No. of patients	SF-12 scoring	*P*	Majeed score	*P*
**Total population**	108	48.22 ± 9.68		83.47 ± 9.23	
**Anterior ring fractures**					
Uunilateral pubic ramus fracture	69	47.93 ± 9.00	0.676	83.22 ± 9.06	0.705
Bilateral pubic ramus fracture	39	48.74 ± 10.88		83.92 ± 9.63	
**Postoperative weight-bearing status**					
Partial Weight-bearing	65	47.89 ± 9.77	0.665	83.18 ± 9.20	0.692
Whole Weight-bearing	43	48.72 ± 13.5		83.91 ± 9.36	
**Age(year)**					
<45	64	51.45 ± 9.38	0.000	86.52 ± 8.12	0.000
≥45	44	43.52 ± 8.12		79.05 ± 9.02	
**ISS score**					
<15	34	48.03 ± 9.13	0.889	83.15 ± 9.34	0.805
≥15	74	48.31 ± 9.97		83.62 ± 9.24	
**Associated injury**					
With lower limb injury	13	42.92 ± 11.32	0.035	77.08 ± 12.41	0.007
Without lower limb injury	95	48.95 ± 9.27		84.35 ± 8.42	

**Notes.**

Statistics and *P*-values were calculated with the *t*-test.

ISS injury severity score SF-12 Short-Form 12

## Discussion

This is the first cases series that focused on isolated anterior pelvic ring fixation for the treatment of LC-1 pelvic fractures. Our study revealed that acceptable functional outcomes can be obtained for LC-1 pelvic fractures with nondisplaced complete sacral fractures by using anterior pelvic ring fixation alone.

Traditionally, it is difficult to evaluate the stability of LC-1 pelvic fractures and determine whether an LC-1 fracture requires operative treatment. Surgeons often have different opinions on the treatment of LC-1 pelvic fractures (Beckmann et al., 2014). Because of unrecognised instability, late displacement of LC-1 fractures treated nonoperatively occurs; therefore, early identification of these unstable injuries might permit earlier surgical intervention to prevent late displacement (Vaidya et al., 2012). In recent years, Sagi et al. formulated a protocol for performing intraoperative radiography to determine fracture stability (Sagi, Coniglione & Stanford, 2011) and developed a strategy using sequential intraoperative examination under anaesthesia (Avilucea et al., 2018). Those studies revealed that some LC-1 fractures show obvious instability. Unfortunately, we did not perform this stress examination to confirm the instability.

Because LC-1 fractures with complete sacral fractures may indicate instability of the pelvic ring (Bruce, Reilly & Sims, 2011; Lefaivre, Padalecki & Starr, 2009), operative stabilisation may be a good choice (Zwingmann et al., 2019). This was the primary reason why we retrospectively analysed patients with complete sacral fractures. Moreover, some studies have reported the use of anterior and posterior fixation for LC-1 fractures (Avilucea et al., 2018; Hagen et al., 2016; Zwingmann et al., 2019). However, we thought that stable fixation of the anterior pelvic ring could change the pattern of LC-1 fractures to that of nondisplaced sacral fractures, which could provide enough support against weight-bearing force. There is no risk of nerve root or blood vessel damage in the posterior approach if fixation of the posterior pelvic ring is not performed. On the basis of the aforementioned reasons, we have used anterior pelvic ring fixation alone for LC-1 pelvic factures with complete sacral fractures in our hospital for the past 5 years.

In our analysis, 15 patients were included in the non-responder group. Those patients were followed up for 19.13 months, but we could not obtain their final function because they either had an excuse (most were too busy) or did not return to the hospital. Therefore, 108 patients were included in the analysis. Functional score results showed that patients with LC-1 pelvic fractures with nondisplaced complete sacral fractures could obtain acceptable clinical results by anterior pelvic ring fixation and conservative treatment of the sacral fractures. According to the confounding factors described in previous studies (Gaffley et al., 2019; Hoffmann, Jones & Sietsema, 2012), we compared the functional outcomes in subgroups by anterior ring fractures, postoperative weight-bearing status, age, ISS, and associated injury. There was no significant functional differences in unilateral and bilateral pubic ramus fractures, in partial and whole weight bearing or ISS <15 and ≥15, between the two subgroups. Age was a confounding factor (Gaffley et al., 2019). The average SF-12 functional scores were 51.45 ± 9.38 and 43.52 ± 8.12 in patients aged >45 years and those ≥45 years, respectively. Moreover, the Majeed scores were 86.52 ± 8.12 and 79.05 ± 9.02 in patients aged <45 years and those ≥45 years, and 62 of 64 patients obtained excellent or good outcomes. Further, a study reported that lower extremity injury is an important factor affecting prognosis (Hoffmann, Jones & Sietsema, 2012). Among our 108 patients, 13 were complicated with lower limb injury with an average SF-12 functional score of 42.92 ± 11.32, whereas 95 without lower limb injury had an average score of 48.95 ± 9.27. The Majeed scores were 77.08 ± 12.41 and 84.35 ± 8.42 in patients with and without lower limb injury, respectively. These data demonstrate that patients’ age and the presence or absence of lower limb injury were important factors for functional recovery, and this finding is consistent with those of previous studies (Gruen et al., 1995; Hoffmann, Jones & Sietsema, 2012), in which young patients with isolated pelvic fractures achieved better function than older patients or those with polytrauma.

Our functional analysis did not emphasise on postoperative plain radiography or the quality of reduction but focused on the function of LC-1 facture patients treated with anterior pelvic ring fixation alone. The relationship between anatomical reduction and the prognosis of pelvic fractures has not been established in current studies (Hessmann et al., 2010; Rommens & Hessmann, 2002). Moreover, all fractures in our study healed by the follow-up, which has indirectly proven the feasibility of anterior pelvic ring fixation alone for treating LC-1 pelvic fractures with nondisplaced complete sacral fractures.

There are some important limitations to this study. Firstly, the study had a retrospective follow-up design, with only an 88% (108/123) response rate, which is low, and we do not know the outcome of 15 patients. Secondly, there was no control group for comparing the main outcome indicators, which makes the findings of this study less convincible and generalisable. Nonetheless, this was the first study to investigate the feasibility of performing only anterior pelvic ring fixation for treating LC-1 pelvic fractures. Our findings may provide a reference for future randomised clinical trials. Thirdly, although there was no significant difference in baseline data between responders and non-responders, information bias could have been introduced by the responders.

## Conclusions

Our results indicate that acceptable functional outcomes can be achieved for LC-1 pelvic fractures with nondisplaced complete sacral fractures by using anterior pelvic ring fixation alone. In future studies, a control group seems necessary to further study the effect of sacral fractures on LC-1 fractures.

##  Supplemental Information

10.7717/peerj.8743/supp-1 Supplemental Information 1 Raw data

## Abbreviations

CT: computed tomography
ISS: injury severity score
LC-1: lateral compression type 1
SF-12: Short Form-12

## Additional Information and Declarations

### Data Availability

The following information was supplied regarding data availability: The raw measurements are available in the Supplementary Files.
